# Public perception of ecosystem services provided by the Mediterranean mussel *Mytilus galloprovincialis* related to anthropogenic activities

**DOI:** 10.7717/peerj.11975

**Published:** 2021-09-15

**Authors:** Veiga Puri, Moreira Juan, Ramos-Oliveira Catarina, Sampaio Leandro, Marcos Rubal

**Affiliations:** 1 CIIMAR Interdisciplinary Centre of Marine and Environmental Research of the University of Porto, Matosinhos, Portugal; 2 Departamento de Biología (Unidad de Zoología) & Centro de Investigación en Biodiversidad y Cambio Global (CIBC-UAM), Universidad Autónoma de Madrid, Madrid, Spain

**Keywords:** Mussels, Ecosystem services, Anthropogenic pressures, Public perception, *Mytilus galloprovincialis*, Public engagement, Marine communities, Portugal

## Abstract

**Background:**

Mussels provide many ecosystem services as habitat, food, water filtration and recreational fishing. However, mussels are vulnerable to anthropogenic pressures such as harvesting or trampling, among others. In this frame, it would be paramount to engage society in marine conservation and improving its awareness about environmental policies. The first step lies in properly assessing what is the perception and concerns of society about marine ecosystems. Our study aims to fill this gap by examining public perception of services provided by *Mytilus galloprovincialis*, its state of conservation and the factors (including anthropogenic activities) shaping mussel beds.

**Methods:**

This study is based on a face-to-face survey consisting of seven open-ended and seven multiple-choice questions of 404 people conducted in 2019 at different shores in the North Portuguese coast. The influence of respondent profile in terms of age, education, gender and place of residence was also assessed.

**Results:**

Most of the participants in our survey (74%) considered that mussels contributed to human well-being and life quality; however, only 31% considered that mussels provide us with many benefits. Regarding the perceived state of mussel services, most of the respondents asserted that mussel services (purification of seawater, habitat, food for other species) worsened in the last 10 years. In contrast, the service as human food was perceived as in an identical state and scientific and traditional knowledge was the only service perceived in a better state. Concerning the state of mussel beds, most of the participants perceived it as good (45%) but a similar percentage (41%) asserted ignoring it. When considering the influence of different factors on mussel beds, only environmental management was considered as having a positive impact by a higher percentage of respondents. The majority of the participants considered that factors included in the questionnaire contributed to worsen mussel beds, ranging between 51% for coastal erosion and 90% for pollution. Education level and age were the main socio-economic factors driving public awareness about the importance of mussel services, its state of conservation and the factors shaping mussel beds.

**Discussion:**

Results showed that perception about the importance of mussels for human well-being and the quantity of delivered benefits increased with the education level. Moreover, older people perceived human food as the most important service offered by mussels. Therefore, our results suggest that mussels are mainly known as food resource; however, most of the people ignore their relevant ecological role and the many other benefits that mussels provide. Thus, it is necessary to actively engage society about importance of mussel beds. As *M. galloprovincialis* is a relevant economic resource, our data could improve the diffusion of knowledge among citizens, stakeholders and scientists, contributing to its sustainability.

## Introduction

Coastal ecosystems provide vital goods and services to humankind (Costanza et al., 1997; Selig et al., 2019). However, in recent decades human activities have dramatically affected and degraded marine ecosystems (Halpern et al., 2008), through the synergistic effects of multiple stressors such as global change, pollution, exploitation of resources or urbanization (Crain, Kroeker & Halpern, 2008; Claudet & Fraschetti, 2010; Todd et al., 2019). Indeed, these stressors have altered ecosystem structure and functioning with consequences to human well-being (De Groot, 1987; Worm et al., 2006). This is particularly evident in intertidal rocky shores because they are easily accessible to humans and thus, experience numerous and diverse threats, that are translated into different impact levels (Thompson, Crowe & Hawkins, 2002). This may, in turn, jeopardize the ability of these ecosystems to provide services and even reduce their amount and value (De Groot, 1987; UNEP, 2006; Nyström et al., 2012).

Many of the dominant species in the rocky intertidal such as macroalgae or bivalves are ecosystem engineers that modify, create or maintain useful habitat for other organisms thus enhancing local biodiversity (Jones, Lawton & Shachak, 1994). Therefore, understanding the role of engineering species is paramount for management of the marine ecosystems because biodiversity loss is considered as one of the most severe global environmental problems (Cardinale et al., 2012; Roe, 2019). The Mediterranean mussel, *Mytilus galloprovincialis* Lamarck 1819, is a widespread ecosystem engineer along the Atlantic rocky shores of the Iberian Peninsula. It is a gregarious species that attaches to rocks or other hard substrates by mean of their byssal threads and plays a relevant role in intertidal food webs (Vinagre et al., 2015). Although less explored than other taxa such as seagrasses or corals (Liquete et al., 2013; Mtwana Nordlund et al., 2016), *M. galloprovincialis* provides many ecosystem services, including support (*e.g.*, habitat for many species, life cycle maintenance, biodiversity), provision of food, regulation of environmental features (water filtration, coastal protection) and cultural services (recreational fishing, tourism) (Beaumont et al., 2007; Montes et al., 2012; Gestoso et al., 2013; Gundersen et al., 2017). This mussel is economically relevant in European countries such as Italy, Spain and Portugal and therefore has long been harvested for food and bait (Rius & Cabral, 2004; Bertocci et al., 2012). In Portugal, this species is particularly relevant as resource in the North due to its proximity with Galicia (Northwest of Spain) where mussels are intensively cultured. However, harvesting results in alteration of the features and extension of mussel beds thus affecting negatively the numbers (species abundance) and variety (total number of species) of the associated biodiversity (Veiga et al., 2019). Although less charismatic than corals or kelps as perceived by people, *M. galloprovincialis* is a species ecologically important and especially vulnerable not only to harvesting but to other anthropogenic pressures such as trampling, presence of non-indigenous species or coastline urbanization (Beukema & Cadée, 1996; Smith & Murray, 2005; Robinson et al., 2007; Carranza et al., 2009; Veiga et al., 2020). In this context, it is mandatory to involve society and improving its awareness about environmental policies to change its behaviour. This let to adopt strategies to achieve the sustainability of marine ecosystems (Jefferson et al., 2014; Ware & Callaway, 2019). However, these strategies have hardly considered evidences about awareness, concerns and priorities of a wide audience (Gelcich et al., 2014). In fact, the first step to engage society in marine conservation is to know their perception about marine ecosystems. Previous studies that have assessed public perception have been focused on valuation of non-monetary goods and services provided by marine ecosystems such as water quality, recreation or wind and tidal energy (Gelcich et al., 2014). Some studies have also evaluated public awareness about anthropogenic impacts on marine ecosystems, mainly those related to global change (*e.g.*, Crona et al., 2013; Chilvers et al., 2014; Shi, Visschers & Siegrist, 2015). Public perception of environmental issues is relevant for adopting successful management options (Jefferson et al., 2015) however, nowadays there are still many gaps on its knowledge (Hawkins et al., 2016) and fairly little effort has been done in the marine realm (Jefferson et al., 2014; Jefferson et al., 2015). Moreover, most studies were done in USA, UK and Australia (Jefferson et al., 2015). Considering that public perception is highly contextual, influenced by many variables such as age, gender, social values, education, place of residence, proximity to the coast or the very country where people live (Crona et al., 2013; Jefferson et al., 2015; Shi, Visschers & Siegrist, 2015), it is necessary to obtain empirical data about public awareness of marine issues not explored yet, embracing different countries and target audiences.

Portugal has one of the European largest Economic Exclusive Zones and is one of the largest maritime nations (Frazão Santos et al., 2014). Moreover, it has a unique geographical location, laying at the intersection between the North Atlantic, the Mediterranean and Western Africa and also shows a wide variety of marine and coastal ecosystems including a long history of seagoing and sea dependency (Oliveira et al., 2013). This makes it an important player in marine matters at the European Union and also higher international levels. Studies mainly done in Portuguese oceanic islands (*i.e.,* Açores, Madeira) have evaluated public awareness of different marine issues such as environmental and socio-economic impacts of artificial reefs, considering different groups of stakeholders (Ramos et al., 2007) and their usefulness to provide ecosystem services but only assessing perception of fishermen (Ramos et al., 2019). Public perception and concern of marine contamination and consumers’ health risk-benefit of seafood was evaluated in five European countries, including Portugal (Jacobs et al., 2015a; Jacobs et al., 2015b). Ressurreição et al. (2012) analyzed the perception of both general population and a group of marine experts in the Açores archipelago concerning drivers of change, pressures and management priorities of marine systems. More recently, Parreti et al. (2020) evaluated baseline knowledge of stakeholders and their perceptions about marine non-indigenous species in Açores and Madeira. Nevertheless, only two studies have focused on continental Portugal (Ramos et al., 2007; Ramos et al., 2019). Therefore, although Portugal is exceptionally well placed to allow for studies demonstrating the value of services provided by marine ecosystems, and incorporating this understanding into marine policies and legal requirements, more studies are needed to improve society engagement in marine topics.

Our study sought to fill this gap by examining public perception of ecosystem services provided by *M. galloprovincialis* and the influence of anthropogenic activities and pressures in Northern Portugal. This was achieved *via* a questionnaire to people visiting coastal localities, consisting of seven open-ended and seven multiple-choice questions. The information obtained, including perception of potential anthropogenic activities on mussels, may help to their management and further improvement of the public understanding of the importance of marine ecosystems for human well-being and economic development.

## Materials & Methods

### Questionnaire design

The questionnaire consisted of 14 questions (seven open-ended and seven multiple-choice) that included 3- or 4-point Likert scale answers and binary. It was subdivided into three sections: (A) perception about ecosystem services provided by mussels (Questions 1-6), (B) factors shaping mussel beds (Questions 7-8) and (C) socio-economic information of the interviewees (Questions 9-14) (Table 1).

**Table 1:** Questionnaire design.

**Q1**. Do you think that mussels contribute in some way to human wellbeing and life quality?		□ Yes *[go to 1.1.]* □ No *[go to 2.]* □ Do not know *[go to 2.]*	
**Q2.** [If yes] How many benefits?		□ Many □ Some □ Few □ None	
**Q3.** [If yes] Can you provide examples of benefits that you consider relevant? *[Write all benefits indicated]*		
**Q4.** From the range of benefits that mussels provide us shown in the panel (Fig. S1) chose three that you consider the most relevant for the wellbeing and life quality of people that live or visit the county (choose only 3 benefits			
**Benefit**	**Q5**. Order by importance*:* *1) Somehow important* *2) Very important* *3) The most important*		**Q6**. In the last 10 years, the benefit is: *1) Worse* *2) Identical* *3) Better* *4) Do not know*
Benefit 1			
Benefit 2			
Benefit 3			

**Q7**. In your opinion, on which condition is the mussel bed on this beach?	□ Good	□ Bad	□ Do not know	
**Q8**. In your opinion, the following factors have a positive impact (improve), a negative impact (worsen), or do not have impact in the mussel beds on this beach?				
***Factor***	***Positive impact***	***Negative impact***	***No impact***	***Not sure***
a. Climate change				
b. Seaside tourism				
c. Environmental management				
d. Pollution				
e. Harvesting of mussels				
f. Coastal erosion				
g. Local fishing				
h. Recreational activities (sport fishing, surf, …)				

**Q9**. What year were you born?				
**Q10**. What is your level of education	□ University □ Secondary education □ Basic education □ None □ Other:			
**Q11**. Are you resident or visitor in this county?	□ Resident	□ Visitor		
**Q12**. Do you consider that you live in an urban or non-urban area?	□ Urban	□ Non-urban		
**Q13.** What is the county where you live most of the time?				
**Q14**. Gender of the interviewee	□ Male	□ Female		

In section A, questions were about benefits provided by mussel beds. The first part of thissection Q1-Q3 aimed to evaluate basic knowledge of public about services provided by mussels. In the second part of A (Q4-Q6), a panel with a brief description of the most important services delivered by mussels (Beaumont et al., 2007; Montes et al., 2012; Gundersen et al., 2017), namely food for humans and other species, purification of seawater, existential value, recreational activity, habitat for other species, ornamentation and scientific and traditional knowledge was shown to interviewees (Table S1). Then participants were asked for selecting the three services from the panel that they considered most important (Q4), ranking them per relevance (Q5) and if the state of the selected services, based on their supply, worsens, improves or remains similar in the last 10 years (Q6).

Section B aimed to know the public perception about the state of mussel beds (Q7) and different factors, including anthropogenic activities that improve, worsen or have no effect on mussel beds (Q8). Finally, the last section (C; Q9-Q14) was designed to include the socio-economic information of the participants: age, gender, education level, if respondents are residents or visitors in the county of the survey and the location of their habitual residence. More details about the location of residency were inferred to address if answers were influenced by the effect of residing in a coastal or non-coastal locality and/or in an urban or non-urban locality. The socio-economic information will be named hereafter as respondent profile.

### Survey collection

**Figure 1:**
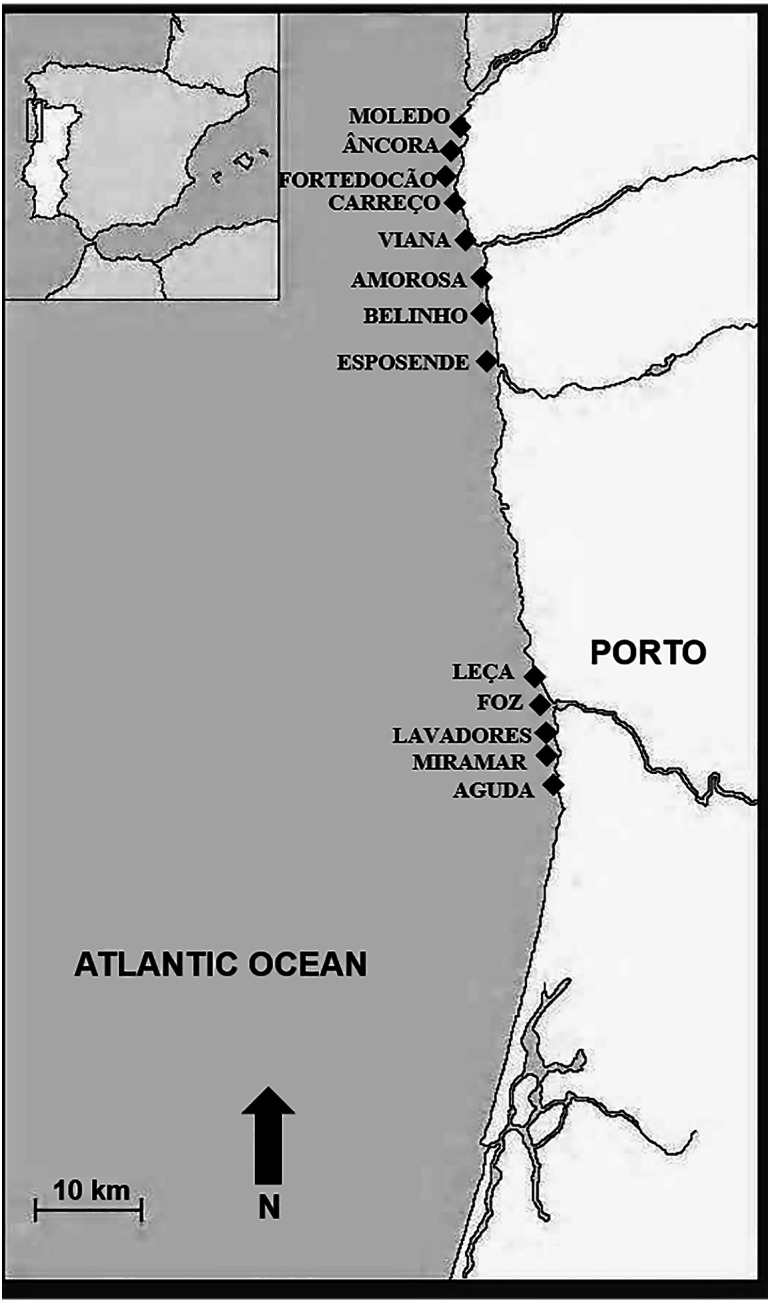
Study area. Location of beaches where interviews were done (northwest Portugal).

A face-to-face survey was undertaken with members of the public visiting 13 different localities in the northern Portuguese coast (Fig. 1) to ascertain their public perception about ecosystem services provided by mussels and those factors (including anthropogenic activities) that influence mussel beds. People were randomly selected to participate in the survey. Interviews were conducted by two different interviewers between 30th March and 24th August 2019; each one lasted generally 7–10 min. A number of 404 interviews were conducted, of which 53% corresponded to females and 47% to males. The age profile of interviewees, their education level and information about their residency place is presented in Table 2. These data were organized in different categories according to the profile of people visiting different localities where interviews were done. Over 45% of interviewees defined themselves as residents and 55% as visitors (Table 2). Moreover, 76% of the respondents considered themselves living in an urban locality and 24% as non-urban whereas 61% lived in coastal localities and 39% in non-coastal ones. Research conducted in this survey was in accordance with the EU Regulation 2016/679 of the European Parliament and of the Council of 27 April 2016 on the protection of natural persons with regard to the processing of personal data and on the free movement of such data (General Data Protection Regulation). Survey was anonymous and prior to starting the questionnaire, those interviewed were provided with an introduction to the activity and gave consent to participate in the study. All participants were informed about their right to refuse to answer any of the questions and to withdraw from participation at any time.

**Table 2:** Respondent profile. Respondent profile of interviewees according to age, education level and place of residency.

**Age group**	**Number**	**%**
Under 40	145	36
40–55	121	30
Over 55	136	34
*N* = 402 (2 not answered)		
**Education level**		
Higher education	156	39
Secondary education	125	31
Basic education	113	28
None	10	2
**Residency**		
Visitor	224	55
Resident	180	45
Urban	306	76
Non-urban	98	24
Coastal	244	61
Non-coastal	159	39
For coastal and non-coastal *N* = 403 (1 not answered)		

### Data analyses

Results from each question of the survey were illustrated by means of figures based on the total number of respondents and percentages. Moreover, Chi-squared tests were used to assess whether there was a relationship between the respondent profiles (*i.e.,* age, sex, education, being resident or visitor, living in an urban/non-urban and coastal/non-coastal locality), and their concern about ecosystem services provided by mussels as well as their perception about factors, including anthropogenic activities influencing mussel beds. Then multinomial logistic regressions (MLR hereafter) were used to identify main variables of respondent profile that determine perception of ecosystem services provided by mussels and factors shaping them. For these regressions, those variables of respondent profile that were significant by Chi-squared tests were used as predictors of answers to different questions included in the questionnaire that were considered as dependent variables. Statistical analyses were done using SPSS (version 26).

## Results

### Services provided by *M. galloprovincialis*

**Figure 2:**
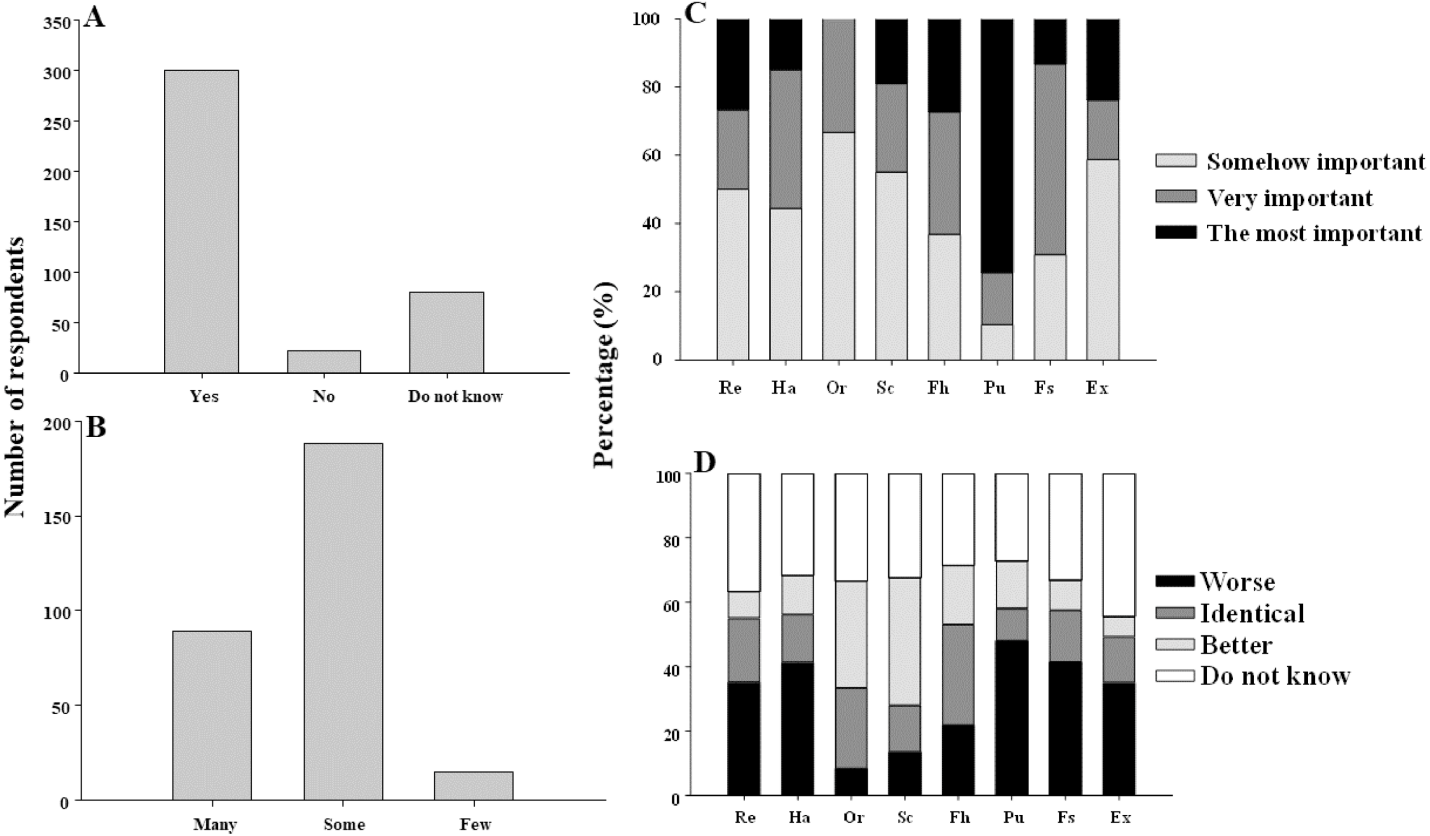
Importance of mussels for human well-being. Perceived importance of mussels for human well-being and life quality (A). Perceived quantity of benefits provided by mussels (B). Results of interviewees asked for selecting the three most important mussel services provided in a list and ordering them in function of their importance. Re, Recreational activity; Ha, Habitat; Or, Ornamentation; Sc, Scientific and traditional knowledge; Fh, Human food; Pu, Purification of seawater; Fs, Food for other species and Ex, Existential value (C). Perceived state of services provided by mussels in the last 10 years (D).

The majority of participants in Q1 (Table 1) considered that mussels contributed in some way to human well-being and life quality (74%, *N* = 300) whereas only 6% (*N* = 22) perceived mussels as not contributing and 20% of the respondents answered not to know if mussels contribute to human life (20%, *N* = 81; Fig. 2A). In terms of the respondent profile, there was only a significant relationship between the perceived importance of mussels and education (*χ*^2^ = 10.882, d.f. = 4, *p* < 0.05; Table S2). MLR pointed out more probable to consider mussels as important to human well-being and life quality among people with university education (Table 3).

**Table 3:** Perceived importance of mussels for human well-being. Results of multinomial logistic regressions assessing the influence of respondent profile on the perceived importance of mussels for human well-being and life quality (Q1) and the quantity of benefits provided by mussels (Q2). Significant differences (*p* < 0.05) indicated in bold.

**Question & Answers**	**Respondent profile**		**Coefficients (*p*-values)**
**Q1**			
No	**Education**	Basic	0.105 (0.849)
		Secondary	−1.338 (0.069)
		University	Reference
Yes		Basic	−0.679 (**0.038**)
		Secondary	−0.567 (0.072)
		University	Reference
Do not know			Reference
**Statistical fit of the model**	Intercept	Final	
−2 Log L	37.219	25.518	
Likelihood ratio test (*p*-value)		11.701 (**0.020**)	
**Q2**			
Many	**Education**	Basic	−2.096 (**0.018**)
		Secondary	−1.330 (0.166)
		University	Reference
	**Age**	<40	−0.241 (0.773)
		40-55	Reference
		>55	0.743 (0.947)
Some	**Education**	Basic	−1.513 (0.080)
		Secondary	−0.361 (0.700)
		University	Reference
	**Age**	<40	0.648 (0.423)
		40–55	Reference
		>55	0.279 (0.698)
Few			Reference
**Statistical fit of the model**	Intercept	Final	
−2 Log L	80.956	57.007	
Likelihood ratio test (*p*-value)		23.949 (**0.02**)	

Among respondents that perceived that mussels contributed to human well-being and life quality, most of them in Q2 (Table 1) considered as well that mussels provided some or many benefits (64%, *N* = 188; 31%, *N* = 89, respectively) whereas only 5% (*N* = 15) considered that they provided few benefits (Fig. 2A). Regarding the effect of respondent profile, there was a significant relationship between the perceived quantity of mussel benefits with age (*χ*^2^ = 9.501, d.f. = 4, *p* < 0.05) and education (*χ*^2^ = 10.882, d.f. = 4, *p* < 0.01) (Table S2). However, MLR only showed a significant influence of education, being more probable to consider that mussels provide many services among people with higher level of education (Table 3).

In order to evaluate the perception about benefits provided by mussels, interviewees were asked (Q3, Table 1) for giving examples of relevant benefits delivered by mussels (Table 4). The most commonly mentioned benefits were food for humans (31%) or other species (26%), improvement of ecosystems (10%) and purification of seawater (10%). Regarding the role as human food, interviewees highlighted their high content in minerals and vitamins and benefits for human health (3%). Other benefits pointed out by comparatively small numbers of interviewees are included in Table 4.

**Table 4:** Benefits provided by mussels. Benefits provided by mussels as pointed by respondents.

**Benefit**	**Number** ^a^	**%**
Food for humans	198	31
Food for other species	167	26
Ecosystem improvement	65	10
Purification of seawater	61	10
High content in minerals and vitamins	21	3
Good for human health	20	3
Habitat	19	3
Increase the biodiversity	15	2
Benefit other species	15	2
Harvesting	11	2
Jobs	11	2
Indicator of water quality	10	2
Tourism enhancement	7	1
Commerce enhancement	6	1
Wellbeing for visitors on the beach	4	1
Ornamentation	3	0
Medical use	2	0
Iodine rich	2	0
Local culture	2	0
Animal wellbeing	2	0
Total	641	

**Notes.**

a Several interviewees cited multiple benefits.

After the first part of the questionnaire, interviewees were asked for choosing the three most relevant services from the range of benefits included in the panel (Table S1), and to rank them in function of their importance for the well-being and life quality of people (Q4, Q5, Table 1). The majority of respondents considered purification of seawater (74%) as the most important service provided by mussels (Fig. 2C). Human food, recreation and existential value were also considered as the most important services by more than 20% of respondents (Fig. 2C). Food for other species, habitat, human food and ornamentation were considered as very important services whereas ornamentation, existential value, scientific and traditional knowledge and recreation were cited as somehow important by most of the respondents (Fig. 2C).

When each service was analyzed separately, we found a significant relationship between responses concerning the importance of mussel harvesting as recreational activity and being resident or visitor (*χ*^2^ = 6.133, d.f. = 2, *p* < 0.05) and living in a coastal or non-coastal locality (*χ*^2^ = 8.114, d.f. = 2, *p* < 0.05) (Table S2). MLR showed more probable to consider mussels as stock for recreational activities as the most important service for people living in coastal localities but there was no influence of whether being resident or visitor (Table 5).

**Table 5:** Perceived importance of different services provided by mussels. Results of multinomial logistic regressions assessing the influence of respondent profile on the perceived importance of different services provided by mussels (Q5) and their state in the last 10 years (Q6). Significant differences (*p* < 0.05) indicated in bold.

**Question & Answers**	**Respondent profile**		**Coefficients (*p*-values)**
**Q5 Recreational activity**			
Somehow important	**Visitor/Resident**	Resident	−0.974 (0.229)
	**Coastal/Non-coastal**	Coastal	0.788 (0.329)
The most important	**Visitor/Resident**	Resident	0.024 (0.980)
	**Coastal/Non-coastal**	Coastal	2.695 (**0.035**)
Very important			Reference
**Statistical fit of the model**	Intercept	Final	
−2 Log L	32.248	19.915	
Likelihood ratio test (*p*-value)		12.333 (**0.015**)	
**Q5 Human food**			
The most important	**Education**	Basic	1.149 (0.091)
		Secondary	−0.201 (0.755)
		University	Reference
	**Age**	<40	1.132 (0.109)
		40–55	Reference
		>55	2.243 (**0.000**)
Very important	**Education**	Basic	0.973 (0.103)
		Secondary	0.277 (0.603)
		University	Reference
	**Age**	<40	0.233 (0.677)
		40–55	Reference
		>55	1.268 (**0.012**)
**Statistical fit of the model**	Intercept	Final	
−2 Log L	80.197	52.179	
Likelihood ratio test (*p*-value)		28.019 (**0.000**)	
**Q6 Recreational activity**			
Better	**Visitor/Resident**	Resident	0.470 (0.700)
Do not know		Resident	−1.833 (**0.007**)
Identical		Resident	−1.386 (0.064)
Worse			Reference
**Statistical fit of the model**	Intercept	Final	
−2 Log L	29.220	18.624	
Likelihood ratio test (*p*-value)		10.596 (**0.014**)	
**Q6 Purification of seawater**			
Better	**Age**	<40	−0.981 (0.125)
		40–55	Reference
		>55	−0.113 (0.843)
Do not know		<40	−0.480 (0.368)
		40–55	Reference
		>55	−1.194 (**0.028**)
Worse		<40	−0.211 (0.679)
		40–55	Reference
		>55	−0.824 (0.105)
Identical			Reference
**Statistical fit of the model**	Intercept	Final	
−2 Log L	58.621	39.616	
Likelihood ratio test (*p*-value)		19.005 (**0.004**)	
**Q6 Food other species**		
Better	**Age**	<40	−0.191 (0.757)
		40–55	Reference
		>55	1.153 (**0.038**)
Do not know		<40	−0.191 (0.566)
		40–55	Reference
		>55	0.092 (0.805)
Identical		<40	−0.037 (0.935)
		40–55	Reference
		>55	0.667 (0.146)
Worse			Reference
**Statistical fit of the model**	Intercept	Final	
−2 Log L	47.531	38.119	
Likelihood ratio test (*p*-value)		9.413 (0.152)	
**Q6 Existential value**			
Better	**Visitor/Resident**	Resident	17.909 (**0.000**)
	**Coastal/Non-coastal**	Coastal	Not calculated
Do not know		Resident	−0.693 (0.431)
		Coastal	−0.365 (0.639)
Identical		Resident	1.386 (0.258)
		Coastal	16.618 (0.995)
Worse			Reference
**Statistical fit of the model**	Intercept	Final	
−2 Log L	41.001	19.559	
Likelihood ratio test (*p*-value)		21402 (**0.002**)	

Concerning mussels as human food, significant relationships between the importance of this service with age (*χ*^2^ = 10.042, d.f. = 4, *p* < 0.05) and education (*χ*^2^ = 14.329, d.f. = 4, *p* < 0.01) of the interviewee were found (Table S2). However, MLR only showed a significant effect of age, being more probable to select this service as the most important or very important among people older than 55 years (Table 5).

About the state of mussel services in the last 10 years (Q6, Table 1), most of the respondents considered that benefits of habitat, purification of seawater and food for other species worsened (41, 48 and 41%, respectively) (Fig. 2D). For recreational activity and existential value, most of the respondents did not know about their state in the last 10 years (37 and 44%, respectively) (Fig. 2D). Mussels as human food were considered as providing the same benefit along the last 10 years by a higher number of interviewees (31%) whereas their scientific and traditional knowledge was the only benefit that respondents considered in a better state (31%) (Fig. 2D). Finally, the same percentage of interviewees answered that ornamentation was in a better state currently or did not know about it (33%) (Fig. 2D).

Results on the perception in the last 10 years for mussel harvesting as recreational activity, showed a significant relationship between its state in the last 10 years and being resident or visitor (*χ*^2^ = 9.900, d.f. = 3, *p* < 0.05; Table S2). MLR pointed out that respondents ignore its state more frequently if they are visitors (Table 5). Regarding ornamentation there was a significant relationship with those living in an urban or non-urban locality (*χ*^2^ = 8.625, d.f. = 3, *p* < 0.05; Table S2); however, MLR was not done for this service because few respondents selected it.

For purification of seawater and mussels as food for other species, there was a significant relationship between the state of these services in the last 10 years and age (*χ*^2^ = 16.146, d.f. = 6, *p* < 0.05; *χ*^2^ = 19.891, d.f. = 6, *p* < 0.01, respectively) (Table S2). MLR showed that is more probable to ignore the state of purification by mussels in people between 40 and 55 years (Table 5). Regarding mussels as food for other species, it is more probable to consider it in a better state among respondents older than 55 years old (Table 5). Finally, for the mussel service of existential value, there was a significant relationship between the state of this service in the last 10 years and being resident or visitor (*χ*^2^ = 17.047, d.f. = 3, *p* < 0.01) and being a coastal or non-coastal locality dweller (*χ*^2^ = 12.147, d.f. = 3, *p* < 0.01; Table S2). However, MLR only found a significant effect of being resident or visitor, with higher probability of considering this service in a better state among residents (Table 5).

### Factors and anthropogenic activities influencing *M. galloprovincialis*

**Figure 3:**
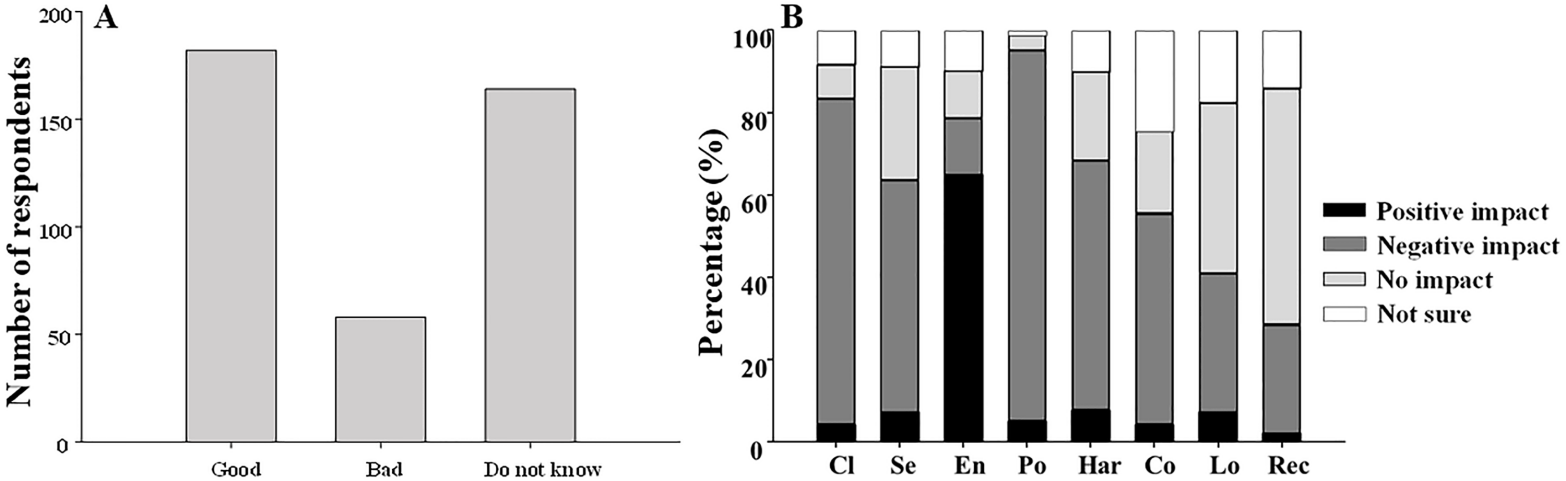
Perceived state of mussel beds. Perceived state of mussel beds (A) and perceived influence of climate change (Cl), seaside tourism (Se), environmental management (En), pollution (Po), harvesting (Ha), coastal erosion (Co), local fishing (Lo) and recreational activities (Rec) on mussel beds (B).

In Q7 (Table 1), the majority of respondents perceived that the condition of mussel beds was good (45%) whereas 41% stated not knowing it and only 14% considered it as bad (Fig. 3A). Regarding the effect of respondent profile in the perceived state of mussel beds, there was only a significant effect of being resident/visitor (*χ*^2^ = 11.313, d.f. = 2, *p* < 0.01) and living in coastal or non-coastal localities (*χ*^2^ = 15.956, d.f. = 2, *p* < 0.01; Table S2). MLR showed that it is more probable to consider the state of mussel beds as bad among residents but the effect of living in coastal or non-coastal localities was not significant (Table 6).

**Table 6:** Perceived condition of mussel beds. Results of multinomial logistic regressions assessing the influence of respondent profile on the perceived condition of mussel beds (Q7) and the influence of the different environmental and anthropogenic factors on mussel beds (Q8). Significant differences (*p* < 0.05) indicated in bold.

**Question & Answers**	**Respondent profile**		**Coefficients (*p*-values)**
**Q7**		
Bad	**Visitor/Resident**	Resident	1.338 **(0.000**)
	**Coastal/Non-coastal**	Coastal	0.379 (0.403)
Good		Resident	0.506 (0.070)
		Coastal	0.303 (0.271)
Do not know			Reference
**Statistical fit of the model**	Intercept		Final
−2 Log L	61.284		33.535
Likelihood ratio test (*p*-value)	27.749 (**0.000**)		
**Q8 Seaside tourism**		
Positive	**Gender**	Female	1.235 (**0.003**)
	**Education**	Basic	1.479 (**0.008**)
		Secondary	0.080 (0.897)
		University	Reference
	**Age**	<40	0.596 (0.292)
		40–55	Reference
		>55	0.038 (0.939)
Negative	**Gender**	Female	0.633 (**0.016**)
	**Education**	Basic	−0.489 (0.145)
		Secondary	−0.468 (0.128)
		University	Reference
	**Age**	<40	0.599 (0.070)
		40–55	Reference
		>55	−0.740 (**0.018**)
Not sure	**Gender**	Female	0.735 (0.078)
	**Education**	Basic	0.168 (0.755)
		Secondary	0.083 (0.866)
		University	Reference
	**Age**	<40	0.200 (0.689)
		40–55	Reference
		>55	−0.891 (0.083)
No impact			Reference
**Statistical fit of the model**	Intercept	Final	
−2 Log L	333.855	180.517	
Likelihood ratio test (*p*-value)		153.338 (**0.000**)	
**Q8 Environmental management**			
Positive	**Gender**	Female	0.366 (0.267)
Negative		Female	0.463 (0.254)
Not sure		Female	1.265 (**0.005**)
No impact			Reference
**Statistical fit of the model**	Intercept	Final	
−2 Log L	37.823	28.929	
Likelihood ratio test (*p*-value)		8.895 (**0.031**)	
**Q8 Mussel harvesting**		
Positive	**Age**	<40	0.629 (0.285)
		40–55	Reference
		>55	0.366 (0.464)
Negative		<40	0.948 (**0.006**)
		40–55	Reference
		>55	−0.479 (0.107)
Not sure		<40	1.545 (**0.007**)
		40–55	Reference
		>55	0.942 (0.073)
No impact			Reference
**Statistical fit of the model**	Intercept	Final	
−2 Log L	70.365	39.313	
Likelihood ratio test (*p*-value)		31.052 (**0.000**)	
**Q8 Recreational activities**			
Positive	Gender	Female	−0.242 (0.749)
	Education	Basic	−0.548 (0.559)
		Secondary	−0.192 (0.826)
		University	Reference
Negative	Gender	Female	0.65 (**0.009**)
	Education	Basic	−0.664 (**0.028**)
		Secondary	−0.573 (**0.049**)
		University	Reference
Not sure	Gender	Female	0.187 (0.545)
	Education	Basic	−1.195 (**0.005**)
		Secondary	−0.501 (0.146)
		University	Reference
No impact			Reference
**Statistical fit of the model**	Intercept	Final	
−2 Log L	89.406	66.307	
Likelihood ratio test (*p*-value)		23.099 (**0.006**)	
**Q8 Coastal erosion**			
Positive	**Gender**	Female	1.044 (0.072)
	**Education**	Basic	−0.819 (0.220)
		Secondary	−1.850 (**0.032**)
		University	Reference
	**Age**	<40	0.474 (0.537)
		40–55	Reference
		>55	0.703 (0.306)
	**Visitor/Resident**	Resident	−0.365 (0.512)
Negative	**Gender**	Female	0.711 (**0.019**)
	**Education**	Basic	−0.745 (**0.049**)
		Secondary	−0.670 (0.066)
		University	Reference
	**Age**	<40	1.072 (**0.006**)
		40–55	Reference
		>55	0.116 (0.740)
	**Visitor/Resident**	Resident	−0.763 (**0.008**)
**Q8 Coastal erosion**			
Not sure	**Gender**	Female	0.532 (0.117)
	**Education**	Basic	0.170 (0.694)
		Secondary	0.242 (0.556)
		University	Reference
	**Age**	<40	0.712 (0.092)
		40–55	Reference
		>55	−0.730 (0.069)
	**Visitor/Resident**	Resident	−0.471 (0.147)
No impact			Reference
**Statistical fit of the model**	Intercept	Final	
−2 Log L	327.861	271.167	
Likelihood ratio test (*p*-value)		56.694 (**0.000**)	
**Q8 Local fishing**			
Positive	**Gender**	Female	0.177 (0.677)
	**Education**	Basic	−0.886 (0.102)
		Secondary	0.076 (0.881)
		University	Reference
	**Age**	<40	−0.277 (0.647)
		40–55	Reference
		>55	0.443 (0.364)
Negative	**Gender**	Female	0.205 (0.428)
	**Education**	Basic	−1.153 (**0.000**)
		Secondary	−0.097 (0.751)
		University	Reference
	**Age**	<40	0.884 (**0.003**)
		40–55	Reference
		>55	−0.505 (0.132)
Not sure	**Gender**	Female	0.519 (0.096)
	**Education**	Basic	−1.308 (**0.003**)
		Secondary	0.132 (0.710)
		University	Reference
	**Age**	<40	1.045 (**0.006**)
		40–55	Reference
		>55	0.230 (0.576)
No impact			Reference
**Statistical fit of the model**	Intercept	Final	
−2 Log L	246.591	172.139	
Likelihood ratio test (*p*-value)		74.452 (**0.000**)	

When interviewees were asked if different factors had a positive, negative or no impact in the mussel beds (Q8, Table 1), the majority of respondents (65%) stated that environmental management had a positive impact on mussel beds. However, pollution (90%), climate change (79%), harvesting of mussels (61%), seaside tourism (56%) and coastal erosion (51%) were considered as factors with a negative impact on mussel beds by the majority of respondents (Fig. 3B). Moreover, recreational activities and local fishing were considered as not having an impact on mussel beds by 57% and 42% of interviewees, respectively, although 34% stated that local fishing also had a negative impact on mussels (Fig. 3B).

When each one of these factors were considered individually, for seaside tourism, a significant relationship between its perceived effect on mussels with sex, age and education level was found (Table S2). MLR showed more probable to consider that seaside tourism has a negative impact among females and people younger than 55 years old and a positive impact among females and people with basic education (Table 6).

Regarding environmental management there was a significant relationship between its perceived effect on mussels and sex (Table S2). MLR showed more probable not to be sure about its effects among females (Table 6).

For harvesting, there was a significant relationship with the age of interviewees (Table S2); being more probable to consider that harvesting has a negative effect on mussels or ignoring its effect among people younger than 40 years old (Table 6).

About recreational activities, there was a significant relationship between its effect on mussels with sex and education (Table S2). MLR showed more probable to consider that it has a negative impact among females and people with a higher level of education (Table 6).

Regarding coastal erosion, there was a significant relationship between its perceived effects on mussels with sex, age, education and being visitor or resident (Table S2). MLR showed more probable to consider that coastal erosion has a negative effect on mussels among females, people younger than 40, with a higher level of education and visitors. Moreover, a positive effect it is more unlikely among people with secondary education (Table 6).

About local fishing, there was a significant relationship between its effect on mussels with sex, age and education (Table S2). MLR showed more probable to consider that local fishing has a negative effect on mussels or not to be sure about its effect among younger people than 40 years old with a higher level of education (Table 6).

## Discussion

Our results provide data of public perception about ecosystem services offered by *Mytilus galloprovincialis*, its state and the factors, including anthropogenic activities shaping mussel beds in Portugal. This mussel plays an important ecological role in the intertidal ecosystems being also a relevant economical resource but less charismatic than other species such as corals (Rius & Cabral, 2004; Vinagre et al., 2015; Mtwana Nordlund et al., 2016). In this way, it could be expected that citizens were less informed about its importance for human welfare. However, most of the participants in our survey considered that mussel beds contributed to human well-being and life quality (Fig. 2A). Internet and television are the principal sources of information for the general public (Gelcich et al., 2014) and can influence people’s perception (Pendleton, Martin & Webster, 2001). In spite of mussels rarely appearing in the media, in news or in documentaries, our data showed that people are familiarized with them because all participants in our survey recognized mussels. This could be explained because humankind has exploited mussels as food resource or bait since ancient times (Oliveira et al., 2013). Although general public is aware that mussels contribute to our well-being, however, only 31% of the respondents considered that mussels provide us with many benefits (Fig. 2B). Moreover, when interviewees were asked for detailing which services mussels offer (Q3, Table 1), most of them pointed out to food resource (57%). This contrasted with their responses after interviewers showed them the panel including different services offered by mussels (Table S1), because at this point of the interview (Q4, Table 1) 74% of the participants in the survey stated that purification of seawater was the most important service offered by mussel beds (Fig. 2C), contrasting with the percentage for this service before seeing the panel (10%). Therefore, this demonstrates that mussels are mainly known because they constitute a food resource. However, most of the people ignore their important ecological role and the many other benefits they provide. In this way, an effort to engage society in understanding about the importance of mussel beds should be done.

Regarding the perceived state of mussel services (Q6, Table 1), most of the participants in this survey asserted that services of purification of seawater, habitat and food for other species were worse in the last 10 years (Fig. 2D). Previous studies have also shown that public perceived the marine environment as deteriorated (Pendleton, Martin & Webster, 2001; Jefferson et al., 2014; Hawkins et al., 2016). In contrast, the service of human food was perceived as in an identical state in the last 10 years and scientific and traditional knowledge was the only service perceived in a better state by a higher percentage of respondents (Fig. 2D).

Public perception is influenced by many variables such as age, gender, education level, social values, socio-economic status, cultural ties, personal experience, place of residence or proximity to the coast (Pendleton, Martin & Webster, 2001; Crona et al., 2013; Chilvers et al., 2014; Jefferson et al., 2015; Shi, Visschers & Siegrist, 2015). When heterogeneous audiences are considered as we did, it is relevant to evaluate if variables as age, gender or social values influence perception (Jefferson et al., 2015). In the first section of the questionnaire (A: Perception about ecosystem services provided by mussels, Table 1), responses were mainly influenced by education and age and in a lower degree by being residents or visitors and coastal or non-coastal dwellers. Our results showed that perception about the importance of mussels for human well-being and the quantity of delivered benefits increased with the education level (Table 3). Gelcich et al. (2014) found that the level of concern about impacts on marine ecosystems was related to the level of acquaintance. Our data seem to point out that people with a higher level of education are more acquainted about the importance of mussel beds. However, Carpenter (2020) in a survey assessing issues related to sea level rise found that education had little impact on responses.

Regarding the effect of age, human food was the most important service among people above 55 years old (Table 5). This age profile also considered that food for other species were in a better state in the last 10 years contrasting with answers given by other age profiles (Table 5). Carpenter (2020) also found that the age influenced responses in a survey concerning sea level rise. Jefferson et al. (2015) emphasized the importance of understanding how age influences the perception, for instance, to conceive specific marine engagement campaigns or effective dissemination and science education programs. In this frame, our data showed that the effort to engage younger and older people about the importance of mussel beds should be different because older people perceived mussels mainly as food resource.

Concerning the influence of the place of residence, previous studies have pointed that people living in coastal areas are more familiarized with marine issues (Fletcher et al., 2009). In concordance, our results also pointed out that mussels as recreational activity, were more important among people living in coastal localities (Table 5). In contrast, visitors ignored its state in a higher proportion (Table 5). This could be explained because many people could be sporadically visiting the locality or even for the first time and therefore, they did not have an earlier reference. Carpenter (2020) also found that perception of vulnerability to natural hazards (*e.g.*, hurricanes, flooding) significantly differed between residents and non-residents; with residents perceiving their communities as more vulnerable than non-residents.

Regarding the state of mussel beds (Q7, Table 1), most of the participants in the survey perceived it as good (45%) but a similar percentage (41%) asserted ignoring it (Fig. 3A). Contrasting results were found about perceived changes in marine water quality in a survey among residents in Los Angeles County. The 58% of the respondents considered that it worsened in comparison to the 20% that considered it in a better state and only 13% did not know about its state (Pendleton, Martin & Webster, 2001). Therefore, our results showed again that it is important to increase the knowledge about mussel beds among public. This response was only influenced by being resident/visitor and coastal/non-coastal dweller; residents considered that mussel beds were in a bad state more frequently than visitors (Table 6). Moreover, visitors and non-coastal dwellers were less aware of the state of mussel beds than residents or coastal dwellers. Similarly, to that reported in the section A of our questionnaire and in previous studies (Fletcher et al., 2009; Carpenter, 2020), residents and people living in coastal localities seem to be better informed about marine topics. In concordance, Gelcich et al. (2014) found that the level of the respondents’ acquaintance and concern on marine impacts increased with the frequency they visited the coast for all marine impacts assessed.

Regarding the influence of different factors on mussel beds in the section B (Q8; Table 1), only environmental management was considered as having a positive impact by a higher percentage of respondents (Fig. 3B). The majority of the participants in the survey considered that most of the factors included in the questionnaire contributed to worsen mussel beds with percentages ranging between 51% for coastal erosion and 90% for pollution (Fig. 3B). Gelcich et al. (2014) also found that pollution (33%), coastal erosion (5%) and climate change (4%) were considered among the main marine impacts. Similarly, Fletcher et al. (2009) found that pollution (41%) and climate change (17.3%) were perceived as the most pressing problems facing the oceans. However, in both previous studies percentages obtained for these factors were lower than those reported in our study. Pendleton, Martin & Webster (2001) found that almost half of the interviewed residents in Los Angeles pointed to water pollution as the main reason for not visiting the beach.

Concerning the influence of the respondent profile in the section B (Perception about factors that influence the mussel beds, Table 1) to the eight factors considered in the questionnaire, the respondent profile was not related to answers regarding environmental management, pollution, climate alterations and local fishing. A plausible explanation could be that people are widely familiarized with these factors (Fletcher et al., 2009; Gelcich et al., 2014) and media coverage could have played an important role (Pendleton, Martin & Webster, 2001; Myatt-Bell et al., 2002).

For the remaining factors, similarly to section A findings, we also found an influence of age, education and being resident/visitor. A higher percentage of participants with a higher level of education perceived that recreational activities, coastal erosion and local fishing had a negative impact on mussel beds whereas those with lower education asserted a positive effect of seaside tourism in a higher percentage (Table 6). A similar pattern was found for age: younger people perceived that seaside tourism, coastal erosion and local fishing had a negative impact whereas older people perceived less frequently their negative effects (Table 6). Semenza et al. (2008) also found that younger people with higher education were more concerned about climate change. Finally, the perception about the impact of coastal erosion was also related to being visitor or resident. In this case, visitors asserted that coastal erosion had a negative effect on mussel beds whereas residents considered that it did not have any effect (Table 6). However, for section B, we also found an influence of gender that it was not reported in the section A of the questionnaire. Females considered that seaside tourism, recreational activities and coastal erosion had a negative impact on mussel beds in a higher percentage than males (Table 6). Stern, Dietz & Kalof (1993) also found that females were more concerned about climate change.

## Conclusions

This study shows how people perceive benefits provided by intertidal beds of the mussel *Mytilus galloprovincialis*, their biological state and factors that influence them in Northern Portugal. Education level, age and place of residence were the main socio-economic factors driving public awareness about importance of mussel beds for human well-being. In this way, our study provides insights for science communication to efficiently raise awareness of the ecosystem services provided by marine systems and the threats marine systems may have been subjected to. Moreover, gaining understanding about how people link to the sea could be useful to properly engage society into marine conservation and resource management. Given that *M. galloprovincialis* is a relevant economic resource, our data and similar future studies in other areas could improve the dissemination and transfer of knowledge among citizens, stakeholders and scientists for contributing to a more efficient management of this resource.

##  Supplemental Information

10.7717/peerj.11975/supp-1 Supplemental Information 1 Panel with a brief description of the most important services delivered by mussels shown to participants in the survey

10.7717/peerj.11975/supp-2 Supplemental Information 2 Chi-square test Results of Chi-square tests assessing the influence of respondent profile on the perceived importance of mussels for human well-being and life quality (Q1), quantity of benefits provided by mussels (Q2), the importance of different ecosystem services provided by mussels (Q5), their state in the last 10 years (Q6), the condition of mussel beds (Q7) and the influence of the different environmental and anthropogenic factors on mussel beds. Significant differences (*p* < 0.05) indicated in bold.

10.7717/peerj.11975/supp-3 Supplemental Information 3 Questionnaire used in the survey

10.7717/peerj.11975/supp-4 Supplemental Information 4 Responses of participants in the survey to different questions

## Additional Information and Declarations

### Data Availability

The following information was supplied regarding data availability: The questionnaire and complete responses are available in the Supplemental Files.
