# Supplementary material for: Public perception of ecosystem services provided by the Mediterranean mussel Mytilus galloprovincialis related to anthropogenic activities
Source: PeerJ. 2021 Sep 15;9:e11975. doi: 10.7717/peerj.11975 (PMC8449534; doi:10.7717/peerj.11975)
Supplement: Supplemental Information 1 [file peerj-09-11975-s001.pdf]

Recreational harvesting

Collection of mussels as recreational activity.

Human food

Mussels are used as food resource in human feeding.

Habitat for other species

Mussels provide particular conditions harbouring many species that live and find protection in mussel beds.

Purification of seawater

Mussels are filter-feeders and thus able to remove pollutants from the seawater improving thus its quality.

Ornamentation

Shells of mussels are used, for instance, in jewellery, handicraft and decoration.

Food for other species

Mussels provide food for other species such as octopus, seastars or birds.

Scientific and traditional knowledge

Scientific and traditional knowledge adquired by the study of mussels.

Existential value

We should have the moral obligation of protecting and conserving species as mussels.
