# Supplementary material for: Public perception of ecosystem services provided by the Mediterranean mussel Mytilus galloprovincialis related to anthropogenic activities
Source: PeerJ. 2021 Sep 15;9:e11975. doi: 10.7717/peerj.11975 (PMC8449534; doi:10.7717/peerj.11975)
Supplement: Supplemental Information 2 — Results of Chi-square tests assessing the influence of respondent profile on the perceived importance of mussels for human well-being and life quality (Q1), quantity of benefits provided by mussels (Q2), the importance of different ecosystem services provided by mussels (Q5), their state in the last 10 years (Q6), the condition of mussel beds (Q7) and the influence of the different environmental and anthropogenic factors on mussel beds. Significant differences (p < 0.05) indicated in bold. [file peerj-09-11975-s002.pdf]

Table S2. Results of Chi-squared tests assessing the influence of respondent profile on the perceived importance of mussels for human well-being and life quality (Q1), quantity of benefits provided by mussels (Q2), the importance of different ecosystem services provided by mussels (Q5), their state in the last 10 years (Q6), the condition of mussel beds (Q7) and the influence of the different environmental and anthropogenic factors on mussel beds. Significant differences (p<0.05) indicated in bold.

Question	Profile	$\chi2$
**Q1**	Sex	$\chi2$ = 0.164, d.f. = 2, p = 0.921
	Age	$\chi2$ = 3.196, d.f. = 4, p = 0.526
	**Education**	$\chi2$ = 10.882, d.f. = 4, **p < 0.05**
	Visitor/Resident	$\chi2$ =5.326, d.f. = 2, p =0.070
	Urban/Non-urban	$\chi2$ = 0.585, d.f. = 2, p = 0.746
	Coastal/Non-coastal	$\chi2$ =4.589, d.f. = 2, p = 0.101
**Q2**	Sex	$\chi2$ = 5.012, d.f. = 2, p = 0.082
	**Age**	$\chi2$ = 9.501, d.f. = 4, **p < 0.05**
	**Education**	$\chi2$ = 14.838, d.f. = 4, **p < 0.01**
	Visitor/Resident	$\chi2$ = 0.210, d.f. = 2, p = 0.901
	Urban/Non-urban	$\chi2$ = 3.442, d.f. = 2, p = 0.179
	Coastal/Non-coastal	$\chi2$ = 0.738, d.f. = 2, p = 0.691
**Q5**		
Harvesting as recreation	Sex	$\chi2$ = 1.312, d.f. = 2, p = 0.519
	Age	$\chi2$ = 4.116, d.f. = 4, p = 0.391
	Education	$\chi2$ = 5.145, d.f. = 4, p = 0.273
	**Visitor/Resident**	$\chi2$ = 6.133, d.f. = 2, **p < 0.05**
	Urban/Non-urban	$\chi2$ = 2.399, d.f. = 2, p = 0.301
	**Coastal/Non-coastal**	$\chi2$ = 8.114, d.f. = 2, **p < 0.05**
Habitat for other species	Sex	$\chi2$ = 0.961, d.f. = 2, p = 0.619
	Age	$\chi2$ = 3.302, d.f. = 4, p = 0.509
	Education	$\chi2$ = 0.174, d.f. = 4, p = 0.996
	Visitor/Resident	$\chi2$ = 2.431, d.f. = 2, p = 0.297
	Urban/Non-urban	$\chi2$ = 0.081, d.f. = 2, p = 0.960
	Coastal/Non-coastal	$\chi2$ = 1.911, d.f. = 2, p = 0.385
Ornamentation	Sex	$\chi2$ = 0.043, d.f. = 1, p = 0.836
	Age	$\chi2$ = 3.225, d.f. = 2, p = 0.199
	Education	$\chi2$ = 0.750, d.f. = 2, p = 0.687
	Visitor/Resident	$\chi2$ = 0.075, d.f. = 1, p = 0.784
	Urban/Non-urban	$\chi2$ = 0.047, d.f. = 1, p = 0.829
	Coastal/Non-coastal	$\chi2$ = 0.500, d.f. = 1, p = 0.480
Scientific and traditional knowledge	Sex	$\chi2$ = 4.428, d.f. = 2, p = 0.109
	Age	$\chi2$ = 3.501, d.f. = 4, p = 0.478
	Education	$\chi2$ = 2.574, d.f. = 4, p = 0.631
	Visitor/Resident	$\chi2$ = 2.163, d.f. = 2, p = 0.339
	Urban/Non-urban	$\chi2$ = 2.408, d.f. = 2, p = 0.300
	Coastal/Non-coastal	$\chi2$ = 2.842, d.f. = 2, p = 0.241
Human food	Sex	$\chi2$ = 2.905, d.f. = 2, p = 0.234
	**Age**	$\chi2$ = 10.042, d.f. = 4, **p <0.05**
	**Education**	$\chi2$ = 14.329, d.f. = 4, **p < 0.01**
	Visitor/Resident	$\chi2$ =0.997, d.f. = 2, p = 0.607
	Urban/Non-urban	$\chi2$ = 0.824, d.f. = 2, p = 0.662
	Coastal/Non-coastal	$\chi2$ = 0.240, d.f. = 2, p = 0.887
Purification of seawater	Sex	$\chi2$ = 1.222, d.f. = 2, p = 0.543
	Age	$\chi2$ = 6.978, d.f. = 4, p = 0.137
	Education	$\chi2$ = 1.611, d.f. = 4, p = 0.807
	Visitor/Resident	$\chi2$ = 2.988, d.f. = 2, p = 0.224
	Urban/Non-urban	$\chi2$ = 2.836, d.f. = 2, p = 0.242
	Coastal/Non-coastal	$\chi2$ = 0.567, d.f. = 2, p = 0.753

**Question**	**Profile**	**$\chi 2$**
**Q5**		
Food for other species	Sex	$\chi 2 = 2.704$, d.f. = 2, p = 0.259
	Age	$\chi 2 = 5.639$, d.f. = 4, p = 0.228
	Education	$\chi 2 = 0.713$, d.f. = 4, p = 0.950
	Visitor/Resident	$\chi 2 = 0.040$, d.f. = 2, p = 0.980
	Urban/Non-urban	$\chi 2 = 1.814$, d.f. = 2, p = 0.404
	Coastal/Non-coastal	$\chi 2 = 0.243$, d.f. = 2, p = 0.886
Existential value	Sex	$\chi 2 = 2.972$, d.f. = 2, p = 0.226
	Age	$\chi 2 = 2.191$, d.f. = 4, p = 0.701
	Education	$\chi 2 = 4.068$, d.f. = 4, p = 0.397
	Visitor/Resident	$\chi 2 = 5.816$, d.f. = 2, p = 0.055
	Urban/Non-urban	$\chi 2 = 1.892$, d.f. = 2, p = 0.388
	Coastal/Non-coastal	$\chi 2 = 0.736$, d.f. = 2, p = 0.692
**Q6**		
Harvesting as recreation	Sex	$\chi 2 =2.932$, d.f. = 3, p = 0.402
	Age	$\chi 2 = 8.168$, d.f. = 6, p = 0.226
	Education	$\chi 2 = 2.641$, d.f. = 6, p = 0.852
	**Visitor/Resident**	$\chi 2 = 9.900$, d.f. = 3, **$p < 0.05$**
	Urban/Non-urban	$\chi 2 = 1.308$, d.f. = 3, p = 0.727
	Coastal/Non-coastal	$\chi 2 = 2.942$, d.f. = 3, p = 0.401
Habitat for other species	Sex	$\chi 2 = 4.872$, d.f. = 3, p = 0.181
	Age	$\chi 2 = 3.402$, d.f. = 6, p = 0.757
	Education	$\chi 2 = 4.502$, d.f. = 6, p = 0.609
	Visitor/Resident	$\chi 2 =7.692$, d.f. = 3, p = 0.053
	Urban/Non-urban	$\chi 2 = 1.313$, d.f. = 3, p = 0.726
	Coastal/Non-coastal	$\chi 2 = 5.984$, d.f. = 3, p = 0.112
Ornamentation	Sex	$\chi 2 = 2.057$, d.f. = 3, p = 0.561
	Age	$\chi 2 = 4.950$, d.f. = 6, p = 0.550
	Education	$\chi 2 = 7.500$, d.f. = 6, p = 0.277
	Visitor/Resident	$\chi 2 = 1.800$, d.f. = 3, p = 0.615
	**Urban/Non-urban**	$\chi 2 = 8.625$, d.f. = 3, **$p < 0.05$**
	Coastal/Non-coastal	$\chi 2 = 4.444$, d.f. = 3, p = 0.217
Scientific and traditional knowledge	Sex	$\chi 2 = 4.068$, d.f. = 3, p = 0.254
	Age	$\chi 2 = 7.742$, d.f. = 6, p = 0.258
	Education	$\chi 2 = 4.148$, d.f. = 6, p = 0.657
	Visitor/Resident	$\chi 2 = 1.458$, d.f. = 3, p = 0.692
	Urban/Non-urban	$\chi 2 = 4.508$, d.f. = 3, p = 0.212
	Coastal/Non-coastal	$\chi 2 = 1.348$, d.f. = 3, p = 0.718
Human food	Sex	$\chi 2 = 3.022$, d.f. = 3, p = 0.388
	Age	$\chi 2 = 4.844$, d.f. = 6, p =0.564
	Education	$\chi 2 = 7.052$, d.f. = 6, p = 0.316
	Visitor/Resident	$\chi 2 = 2.962$, d.f. = 3, p = 0.398
	Urban/Non-urban	$\chi 2 = 3.341$, d.f. = 3, p = 0.342
	Coastal/Non-coastal	$\chi 2 = 2.371$, d.f. = 3, p = 0.499
Purification of seawater	Sex	$\chi 2 = 1.225$, d.f. = 3, p = 0.747
	**Age**	$\chi 2 = 16.146$, d.f. = 6, **$p < 0.05$**
	Education	$\chi 2 = 3.579$, d.f. = 6, p = 0.733
	Visitor/Resident	$\chi 2 = 4.245$, d.f. = 3, p = 0.236
	Urban/Non-urban	$\chi 2 = 2.292$, d.f. = 3, p = 0.514
	Coastal/Non-coastal	$\chi 2 = 3.636$, d.f. = 3, p = 0.303
Food for other species	Sex	$\chi 2 = 2.211$, d.f. = 3, p = 0.530
	**Age**	$\chi 2 = 19.891$, d.f. = 6, **$p < 0.01$**
	Education	$\chi 2 = 10.624$, d.f. = 6, p = 0.101
	Visitor/Resident	$\chi 2 = 3.488$, d.f. = 3, p = 0.322
	Urban/Non-urban	$\chi 2 = 3.876$, d.f. = 3, p = 0.275
	Coastal/Non-coastal	$\chi 2 = 4.510$, d.f. = 3, p = 0.211

Question	Profile	$\chi 2$
**Q6**		
Existential value	Sex	$\chi 2 = 2.266$, d.f. = 3, $p = 0.519$
	Age	$\chi 2 = 10.288$, d.f. = 6, $p = 0.113$
	Education	$\chi 2 = 7.002$, d.f. = 6, $p = 0.321$
	**Visitor/Resident**	$\chi 2 = 17.047$, d.f. = 3, $\mathbf{p < 0.01}$
	Urban/Non-urban	$\chi 2 = 4.749$, d.f. = 3, $p = 0.191$
	**Coastal/Non-coastal**	$\chi 2 = 12.147$, d.f. = 3, $\mathbf{p < 0.01}$
**Q7**	Sex	$\chi 2 = 1.321$, d.f. = 2, $p = 0.517$
	Age	$\chi 2 = 5.881$, d.f. = 4, $p = 0.208$
	Education	$\chi 2 = 6.209$, d.f. = 4, $p = 0.184$
	**Visitor/Resident**	$\chi 2 = 11.313$, d.f. = 2, $\mathbf{p < 0.01}$
	Urban/Non-urban	$\chi 2 = 3.316$, d.f. = 2, $p = 0.191$
	**Coastal/Non-coastal**	$\chi 2 = 15.956$, d.f. = 2, $\mathbf{p < 0.01}$
**Q8**		
Climate alterations	Sex	$\chi 2 = 5.251$, d.f. = 3, $p = 0.154$
	Age	$\chi 2 = 7.559$, d.f. = 6, $p = 0.272$
	Education	$\chi 2 = 8.391$, d.f. = 6, $p = 0.211$
	Visitor/Resident	$\chi 2 = 2.593$, d.f. = 3, $p =0.458$
	Urban/Non-urban	$\chi 2 = 0.855$, d.f. = 3, $p = 0.836$
	Coastal/Non-coastal	$\chi 2 = 1.843$, d.f. = 3, $p = 0.606$
Seaside tourism	**Sex**	$\chi 2 = 9.066$, d.f. = 3, $\mathbf{p < 0.05}$
	**Age**	$\chi 2 = 29.297$, d.f. = 6, $\mathbf{p < 0.001}$
	**Education**	$\chi 2 = 28.860$, d.f. = 6, $\mathbf{p < 0.001}$
	Visitor/Resident	$\chi 2 = 4.488$, d.f. = 3, $p = 0.213$
	Urban/Non-urban	$\chi 2 = 1.168$, d.f. = 3, $p = 0.761$
	Coastal/Non-coastal	$\chi 2 = 2.583$, d.f. = 3, $p = 0.460$
Environmental management	**Sex**	$\chi 2 = 9.589$, d.f. = 3, $\mathbf{p < 0.05}$
	Age	$\chi 2 = 1.354$, d.f. = 6, $p = 0.969$
	Education	$\chi 2 = 7.900$, d.f. = 6, $p = 0.246$
	Visitor/Resident	$\chi 2 = 2.226$, d.f. = 3, $p = 0.527$
	Urban/Non-urban	$\chi 2 = 1.764$, d.f. = 3, $p = 0.623$
	Coastal/Non-coastal	$\chi 2 = 1.545$, d.f. = 3, $p = 0.672$
Pollution	Sex	$\chi 2 = 5.321$, d.f. = 3, $p = 0.150$
	Age	$\chi 2 = 11.044$, d.f. = 6, $p = 0.087$
	Education	$\chi 2 = 6.874$, d.f. = 6, $p = 0.333$
	Visitor/Resident	$\chi 2 = 7.544$, d.f. = 3, $p = 0.056$
	Urban/Non-urban	$\chi 2 = 2.866$, d.f. = 3, $p = 0.413$
	Coastal/Non-coastal	$\chi 2 = 0.439$, d.f. = 3, $p = 0.932$
Harvesting	Sex	$\chi 2 = 5.252$, d.f. = 3, $p = 0.154$
	**Age**	$\chi 2 = 17.660$, d.f. = 6, $\mathbf{p < 0.01}$
	Education	$\chi 2 = 7.198$, d.f. = 6, $p = 0.303$
	Visitor/Resident	$\chi 2 = 2.806$, d.f. = 3, $p = 0.423$
	Urban/Non-urban	$\chi 2 = 2.968$, d.f. = 3, $p = 0.397$
	Coastal/Non-coastal	$\chi 2 = 2.946$, d.f. = 3, $p = 0.400$
Coastal erosion	**Sex**	$\chi 2 = 11.182$, d.f. = 3, $\mathbf{p < 0.05}$
	**Age**	$\chi 2 = 20.720$, d.f. = 6, $\mathbf{p < 0.01}$
	**Education**	$\chi 2 = 18.158$, d.f. = 6, $\mathbf{p < 0.01}$
	**Visitor/Resident**	$\chi 2 = 9.561$, d.f. = 3, $\mathbf{p < 0.05}$
	Urban/Non-urban	$\chi 2 = 5.289$, d.f. = 3, $p = 0.152$
	Coastal/Non-coastal	$\chi 2 = 1.602$, d.f. = 3, $p = 0.659$
Local fishing	**Sex**	$\chi 2 = 11.736$, d.f. = 3, $\mathbf{p < 0.01}$
	**Age**	$\chi 2 = 47.376$, d.f. = 6, $\mathbf{p < 0.01}$
	**Education**	$\chi 2 = 41.234$, d.f. = 6, $\mathbf{p < 0.01}$
	Visitor/Resident	$\chi 2 = 5.984$, d.f. = 3, $p = 0.112$
	Urban/Non-urban	$\chi 2 = 6.77$, d.f. = 3, $p = 0.080$
	Coastal/Non-coastal	$\chi 2 = 0.0684$, d.f. = 3, $p = 0.995$

**Question**	**Profile**	**$\chi2$**
**Q8**		
Recreational activities	**Sex**	$\chi2$ = 11.965, d.f. = 3, **$p < 0.01$**
	Age	$\chi2$ = 10.738, d.f. = 6, p = 0.097
	**Education**	$\chi2$ = 17.585, d.f. = 6, **$p < 0.01$**
	Visitor/Resident	$\chi2$ = 1.340, d.f. = 3, p = 0.720
	Urban/Non-urban	$\chi2$ = 5.906, d.f. = 3, p = 0.116
	Coastal/Non-coastal	$\chi2$ = 3.724, d.f. = 3, p = 0.293
