# Supplementary material for: Public perception of ecosystem services provided by the Mediterranean mussel Mytilus galloprovincialis related to anthropogenic activities
Source: PeerJ. 2021 Sep 15;9:e11975. doi: 10.7717/peerj.11975 (PMC8449534; doi:10.7717/peerj.11975)
Supplement: Supplemental Information 3 [file peerj-09-11975-s003.pdf]

Portuguese version

Uma equipa do Centro Interdisciplinar de Investigação Marinha e Ambiental do Porto está a fazer um estudo sobre a contribuição dos mexilhões para o bem-estar e qualidade de vida das pessoas. Para isso, temos entrevistado pessoas que frequentam praias do norte de Portugal. Este estudo servirá para melhorar o conhecimento científico das percepções do público sobre os mexilhões. Este inquérito é anónimo e voluntário, e não existem respostas certas nem erradas, apenas queremos saber a sua opinião.

SECÇÃO A. Percepção sobre os serviços do ecossistema dos mexilhões

As próximas perguntas são sobre os benefícios que todos nós obtemos da natureza. Estes benefícios são essenciais para o nosso bem-estar e qualidade de vida.

**1.** Acha que os mexilhões contribuem de alguma forma para o bem-estar e qualidade de vida das pessoas?	☐ Sim *[ir para 1.1.]*<br>☐ Não *[ir para 2.]*<br>☐ Não sei *[ir para 2.]*
**1.1.** *[Se sim]* Quantos benefícios?	☐ Muitos ☐ Alguns<br>☐ Poucos ☐ Nenhum
**1.2.** *[Se sim]* Pode dar exemplos de benefícios que considera importantes?<br><br>*[Apontar todos os benefícios indicados]*	

[Se for mencionado um serviço do ecossistema válido na pergunta 1.2. que não aparece no painel de serviços do inquérito, considerar como opção para a pergunta 2.]

2. *[Mostrar painel]* Deste conjunto de benefícios que os mexilhões nos prestam, escolha aqueles que considera serem os ***mais importantes*** para o bem-estar e qualidade de vida das pessoas que visitam esta praia. *[escolher apenas **3 benefícios**]*

**Benefício**	**2.1.** Ordene **por importância**:<br><br>*1) Algo importante*<br>*2) Muito importante*<br>*3) O mais importante*	**2.2.** Nos **últimos 10 anos**, *[o benefício]* está:<br><br>*1) Pior*<br>*2) Igual*<br>*3) Melhor*<br>*4) Não sabe*
1º		
2º		
3º		

SECÇÃO B. Percepção sobre os factores que afectam as comunidades de mexilhão

**3.** Na sua opinião, em que condição se encontra a comunidade de mexilhão desta praia?	☐ Boa ☐ Má ☐ Não sabe

4. Na sua opinião, os seguintes factores melhoram, pioram, ou não têm impacto nenhum na comunidade de mexilhão desta praia?

Factor	***Melhoram***	***Pioram***	***Nenhum impacto***	***Não tem certeza***
Alterações climáticas				
Turismo balnear				
Gestão ambiental				
Poluição				
Apanha de mexilhão				
Erosão costeira				
Pesca local				
Act. Recreio (pesca desportiva, surf, etc.)				

SECÇÃO C. Informação socio-económica

Para finalizar, gostaria de saber…

**5.** É residente ou está de visita?	☐ Residente ☐ Visitante
**6.** Em que ano nasceu?	
**7.** Qual é o seu grau de escolaridade?	☐ Ensino Superior<br>☐ Ensino Secundário<br>☐ Ensino Básico<br>☐ Nenhum<br>☐ Outro
**8.** Considera que vive numa zona urbana ou não urbana?	☐ Urbana ☐ Não urbana

Muito obrigado por ter colaborado e participado neste inquérito. As suas respostas são muito importantes para o nosso estudo. Caso necessite de algum esclarecimento adicional, não hesite em contactar-nos. *[Deixar contacto]*

[A completar pelo/a entrevistador/a]

**9.** Género do/a entrevistado/a	☐ M ☐ F
**10.** Atitude do/a entrevistado/a	☐ Boa ☐ Por vezes interessado/a<br>☐ Desinteressado/a
**11.** Nível de compreensão do inquérito	☐ Elevado ☐ Médio ☐ Baixo
**12.** Local da entrevista (praia)	
**13.** Data	
**14.** Entrevistador/a	

English version

A team of the Interdisciplinary Centre of Marine and Environmental of the University of Porto is doing a study about contribution of mussels for wellbeing and life quality of people. To achieve this, we are interviewing people visiting beaches in the North of Portugal. This study will improve scientific knowledge on public perception about mussels. This survey is anonymous and voluntary, and there are not right and wrong answers, we only want to know your opinion.

Section A. Perception about ecosystem services provided by mussels

Next questions are about benefits that we obtain from nature. These benefits are essential for our wellbeing and quality of life.

**1.** Do you think that mussels contribute in some way to human wellbeing and life quality?	☐ Yes *[go to 1.1.]*<br>☐ No *[go to 2.]*<br>☐ Do not know *[go to 2.]*
**1.1** *[If yes]* How many benefits?	☐ Many ☐ Some<br>☐ Few ☐ None
**1.2** *[If yes]* Can you provide examples of benefits that you consider relevant?<br><br>*[Write all benefits indicated]*	

[In case a valid ecosystem service is mentioned as a response to question 1.2 that is not included in the interview services panel, please consider it as an option for question 2.]

2. *[Show panel]* From this range of benefits that mussels provide us, chose three that you consider the **most relevant** for the wellbeing and life quality of people that live and visit the county (choose only **3 benefits**).

**Benefit**	**2.1** Order by importance:<br><br>*1) Somehow important*<br>*2) Very important*<br>*3) The most important*	**2.2** In the last 10 years, (the benefit) is:<br><br>*1) Worse*<br>*2) Identical*<br>*3) Better*<br>*4) Do not know*
1st		
2nd		
3rd		

Section B. Perception about factors that influence the mussel community

**3** In your opinion, on which condition is the mussel community on this beach?	☐ Good ☐ Bad ☐ Do not know

4 In your opinion, the following factors have a positive impact (improve), a negative impact (worse), or do not have impact in the mussel community on this beach?

Factor	***Positive impact***	***Negative impact***	***No impact***	***Not sure***
Climatic alterations				
Seaside tourism				
Environmental management				
Pollution				
Harvesting of mussels				
Coastal erosion				
Local fishing				
Recreational activities (sport fishing, surf, ...)				

Section C. Socio-economic information

To finish, I would like to know…

**5** Are you resident or visitor?	☐ Resident ☐ Visitor
**6** What year were you born?	
**7** What is your level of education/scholarship?	☐ Higher education<br>☐ Secondary education<br>☐ Basic education<br>☐ None<br>☐ Other:
**8** Do you consider that you live in an urban or non-urban area?	☐ Urban ☐ Non-urban

Thank you very much for your collaboration and participation in this inquiry. Your opinion is very important for our study. In case you need a further enlighten do not hesitate to contact us. *[Leave contact]*

[To be completed by the interviewer]

**9** Gender of the interviewee	☐ M ☐ F
**10** Attitude of the interviewee	☐ Good ☐ Sometimes interested<br>☐ Not interested
**11** Level of understanding of the inquiry	☐ High ☐ Medium ☐ Low
**12** Interview location	
**13** Date	
**14** Interviewer	
